# 136. Impact of a Tele-Stewardship and Tele-Infection Prevention Program on Hospital-Onset Clostridioides Difficile Rates in a Community Three-Hospital Health System

**DOI:** 10.1093/ofid/ofad500.209

**Published:** 2023-11-27

**Authors:** Matthew R Davis, Alex Trzebucki, Erin K McCreary, Nupur Gupta, Templer Suzanne

**Affiliations:** Infectious Diseases Connect; UPMC, Austin, Texas; Department of Medicine / University of Pittsburgh School of Medicine, Pittsburgh, PA; UPMC, Pittsburgh, PA; UPMC, Pittsburgh, PA; University of Pittsburgh, Pittsburgh, Pennsylvania

## Abstract

**Background:**

Clostridioides difficile infection (CDI) is a common hospital-acquired infection (HAI) that can be difficult to diagnose accurately given the numerous test modalities with differing performance characteristics. Inaccurate diagnosis of CDI can negatively affect patient care decisions as well as reimbursement rates. Community and rural hospitals (CARH) may lack specialized personnel or knowledge to implement diagnostic best practices, but telehealth solutions like tele-infection prevention (TIP) and tele-antimicrobial stewardship (TASP) can provide specialist support for CARHs to optimize diagnostic pathways, improve accuracy, and reduce unnecessary treatment.

**Methods:**

In June 2021 under the guidance of TIP and TASP recommendations, *C. difficile* testing was transitioned from a 1-step nucleic acid amplification test (NAAT)-only algorithm to a 2-step algorithm that included NAAT and toxin enzyme immunoassay (EIA) testing. A 6-month pre- and post-implementation assessment was conducted to assess the impacts of this change in hospital-onset CDI (HO-CDI) case rate. Additionally, total *C. difficile* (community- and hospital-onset CDI) rates per 1000 patient days (PD) and consumption of CDI therapeutics were assessed. For statistical analysis, Wilcoxon ranked-sum test to assess changes in CDI rates. Estimated annualized cost-savings for therapeutics were calculated based on institutional purchasing costs.

**Results:**

Following implementation, a 35.9% reduction in HO-CDI was observed (pre 0.96 cases/1000PD vs. 0.61 cases/1000PD; *p* = 0.054). A 10.2% reduction was observed in total CDI (pre 3.66 cases/1000PD vs. 3.28 cases/1000PD; *p* = 0.37). Additionally, a 30.6% reduction was observed in consumption of CDI therapeutics (20.1 doses/1000PD vs. 14.0 doses/1000PD) resulting in an annualized cost-savings of approximately $9200.

**Conclusion:**

Our TIP and TASP advised a community three-hospital health system on optimal diagnostics resulting in a change in their algorithm. Following this, considerable reductions were observed in HO-CDI, total CDI, consumption of CDI therapeutics, and the associated costs of care.

**Disclosures:**

**Erin K. McCreary, PharmD**, Abbvie: Advisor/Consultant|Ferring: Advisor/Consultant|GSK: Honoraria|La Jolla (Entasis): Advisor/Consultant|LabSimply: Advisor/Consultant|Merck: Advisor/Consultant|Shionogi: Advisor/Consultant|Shionogi: Honoraria

